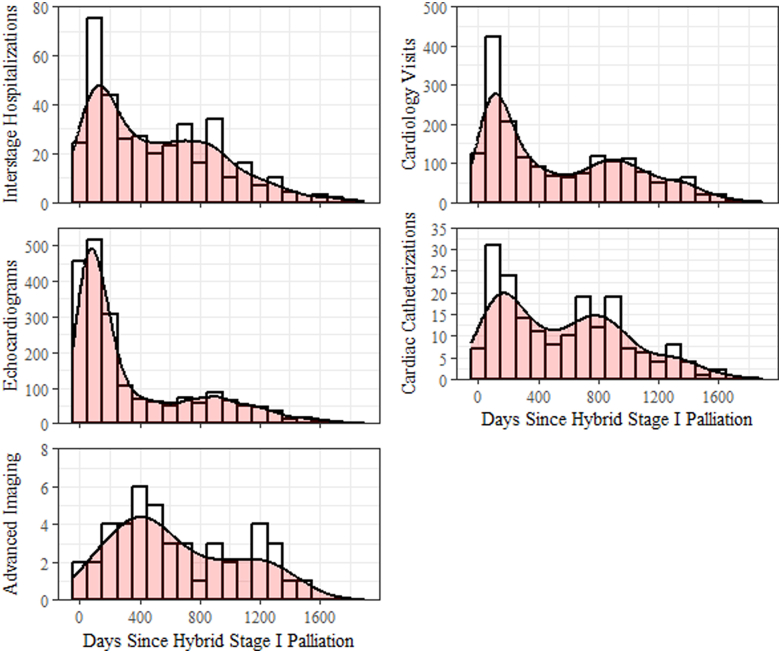# Efficiency at Heart: Navigating the Hybrid Single-Ventricle Pathway

**DOI:** 10.1016/j.atssr.2024.02.017

**Published:** 2024-03-23

**Authors:** Dariya Hardisky, Divyaam Satija, Karen Texter, Chance Alvarado, Mark Galantowicz, Sergio A. Carrillo

**Affiliations:** 1 Department of Surgery, The Ohio State University, Columbus, Ohio; 2 The Ohio State University College of Medicine, Columbus, Ohio; 3 Division of Cardiology, Department of Pediatrics, Nationwide Children’s Hospital, Columbus, Ohio; 4 Biostatistics Resource, Abigail Wexner Research Institute, Nationwide Children’s Hospital, Columbus, Ohio; 5 Department of Cardiothoracic Surgery, Nationwide Children’s Hospital, Columbus, Ohio

## Abstract

**Background:**

Single-ventricle cardiac defects (SVCDs) are among of the most health care resource–intensive congenital diseases. Although SVCDs are traditionally palliated using the Norwood pathway, in the last 2 decades select programs have used the hybrid strategy, which redistributes the operative and interstage risks. This study sought to characterize resource use for a cohort of patients with hybrid-palliated SVCD.

**Methods:**

All patients with SVCDs who underwent palliation with the hybrid strategy and who were followed up exclusively at our institution from January 2008 to December 2021 were included. End points were death, Norwood conversion, orthotopic heart transplantation, 6 months post-Fontan status, or 4 years of age in those patients who had not completed staged palliation. Primary end points included total days hospitalized, number of cardiology visits, echocardiograms, catheterizations, and advanced imaging performed.

**Results:**

Of 135 patients with a diagnosis of SVCD, 72 survived for 6 months after the Fontan procedure. By patient-year for the entirety of the pathway, patients had a median hospital length of stay of 16 days (interquartile range [IQR], 12-25 days), 7 cardiology visits (IQR, 6-9), 8 echocardiograms (IQR, 7-10), and 0.7 catheterizations (IQR, 0.4-1.1). The interstage 1 period had the heaviest resource burden requiring intense cardiology follow-up and echocardiography surveillance. Cardiac catheterizations and advanced imaging were most prevalent during interstage 2 period, with a median of 2 (IQR, 1-2) catheterizations and 36 (40%) patients undergoing advanced imaging. The total median number of hospital days per patient was 63 days (IQR, 47-98.5 days).

**Conclusions:**

Resource use for the care of patients with SVCDs is significant. The intensity of surveillance decreases as patients progress through the pathway. In comparison with published Norwood pathway data, resource intensity and use patterns in hybrid palliation are comparable.

## Visual Abstract

**Figure:**
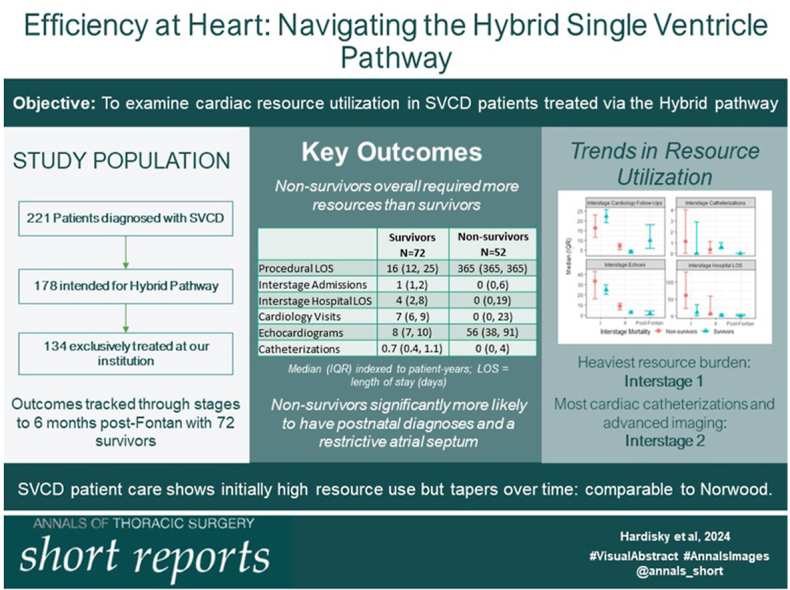

**In Short**

▪ The hybrid strategy for patients with single-ventricle cardiac defects, studied from 2008 to 2021, shows significant resource use: median 19.3 hospital days, 7.5 cardiology visits, 5.3 echocardiograms, and 0.8 catheterizations per patient-year.
▪ Most resources were required during the interstage 1 period, with high needs for cardiology follow-up and echocardiography surveillance.
▪ Compared with the Norwood pathway, hybrid palliation demonstrates comparable patterns in resource intensity and use.

Single-ventricle cardiac defects (SVCDs) are among of the most challenging and resource-intensive cardiac anomalies, making up 3% of all congenital heart diseases.^1^ Advances in surgical techniques and critical care have improved survival, yet there are limited data on the health care resource burden associated with SVCD palliation.^2^

The hybrid palliation pathway integrates catheter-based and surgical methods and aims to avoid extensive neonatal surgical reconstruction. The hybrid approach redistributes operative and interstage risks compared with the traditional Norwood pathway, and thus, understanding patterns of resource use is crucial.^3^^,^^4^ This knowledge could be useful for heart teams in the allocation of services, as well as for parental counseling. To fill the paucity of data on the hybrid palliation burden, our study aimed to examine health care resource use in patients treated exclusively with the hybrid pathway, thereby providing new insights into this established approach in comparison with the traditional Norwood pathway.

## Patients and Methods

This was a single-center, retrospective cohort study performed at a quaternary children’s hospital. The electronic institutional database was queried for patients with a diagnosis of SVCD who were born between January 2008 and December 2021 and who were treated with the hybrid pathway as the primary intent and followed up exclusively at our institution until death, Norwood conversion, orthotopic heart transplantation, 6 months post-Fontan status, or 4 years of age. Institutional Review Board approval was received before study initiation (STUDY00003086, approved 02/14/2023), including a waiver of parental consent.

Resource use was detailed through hospital length of stay (LOS), cardiology visits, echocardiograms, cardiac computed tomography angiography, cardiac magnetic resonance imaging, and cardiac catheterization procedures. Primary end points were indexed in patient-years to allow for comparison between survivors and nonsurvivors, with the percentage of patients reported for low-prevalence end points. Analyzed risk factors included the following: (1) prematurity <37 weeks’ gestation, (2) birth weight <2500 g, (3) postnatal diagnosis, (4) the presence of a genetic syndrome, (5) high-risk cardiac morphologic features, and (6) extracardiac congenital anomalies.

Demographics and resource use metrics were presented as either median (interquartile range [IQR]) or count (%). Resource use metrics comparisons between survivors and nonsurvivors used the Wilcoxon rank sum test. All statistical tests were 2-tailed, and *P* < .05 was considered statistically significant. Univariable negative binomial regression assessed potential risk factors, with an offset term for individual contribution time. All statistical analyses were performed using R software version 4.2.2 (R Foundation).

## Results

**Figure 1:**
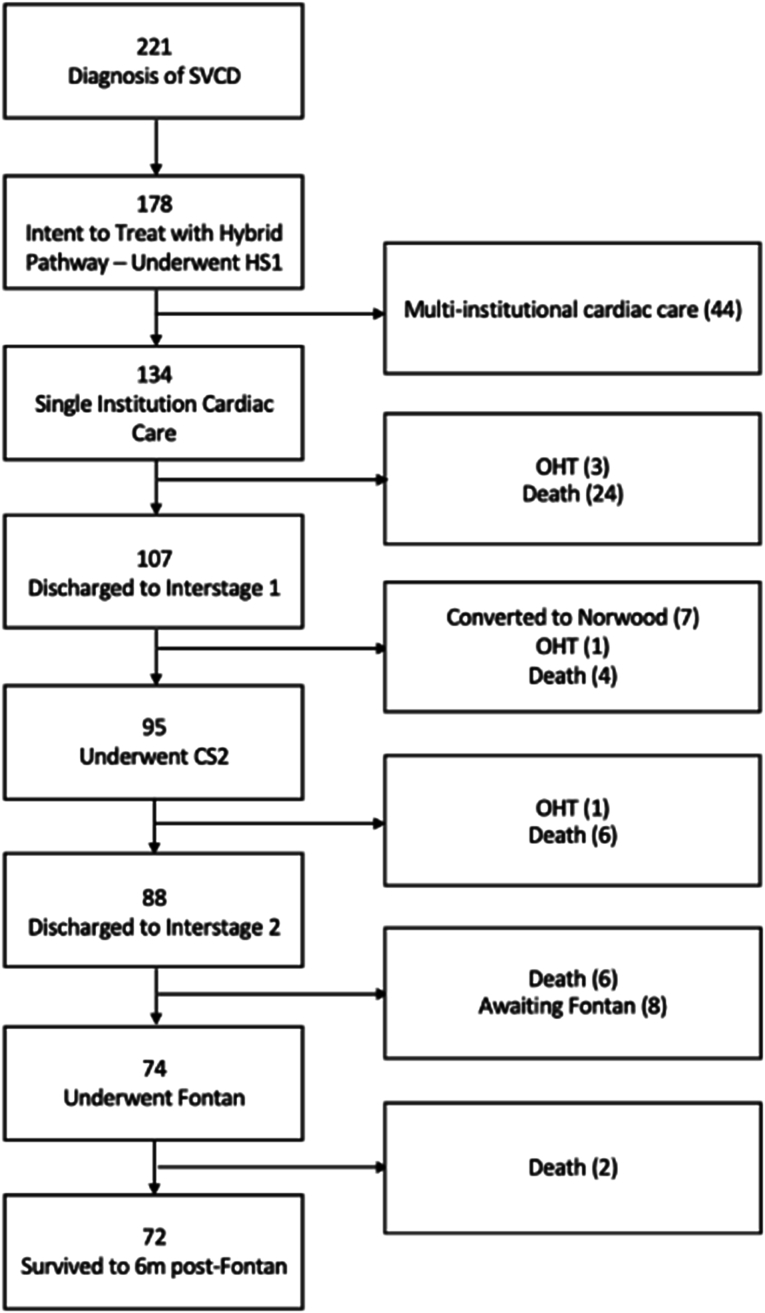
Consolidated Standards of Reporting Trials (CONSORT) diagram of 221 patients with a diagnosis of single-ventricle cardiac defect (SVCD). (CS2, comprehensive stage 2; HS1, hybrid stage 1; OHT, orthotopic heart transplantation.)

A total of 221 patients with SVCDs were treated at our institution between January 2008 and December 2021. Of those patients, 135 (61%) underwent hybrid stage 1 (HS1) procedures with primary intent and received care exclusively at our institution, with 72 (53%) patients reaching transplant-free survival at 6 months after the Fontan procedure. An additional 7 (5%) patients were converted to Norwood procedures during interstage 1 (IS1), and 8 (6%) were awaiting Fontan procedures (Figure 1). The cohort was predominantly male (83; 61%), with 13 (9.6%) premature, 13 (9.6%) with low birth weight, and 20 (15%) with genetic syndromes. Dominant right ventricular morphologic features were present in 93% (n = 124) of cases, mostly hypoplastic left heart syndrome (HLHS; n = 96; 72%) (Table 1).

**Table 1:** Cohort Demographics^a^

Characteristic	Values N = 135
Sex	
Male	83 (61)
Female	51 (39)
Gestational age, d	273 (266-274)
Premature (<37 wk)	13 (9.6)
Birth weight, kg	3.22 (2.91-3.60)
Low birth weight (<2500 g)	13 (9.6)
Postnatal diagnosis	28 (21)
Genetic syndrome	19 (14)
Extracardiac anomaly	19 (14)
SVCD diagnosis	
HLHS	96 (72)
AA/MA	32
AA/MS	26
AS/MS	36
AS/MA	2
DILV	7 (5.3)
DORV with MA	7 (5.3)
Unbalanced AV canal	11 (8.3)
TA	2 (1.5)
Other	10 (7.5)
Dominant ventricle	
Left	9 (6.8)
Right	124 (93)
High-risk cardiac anatomy	
Ascending aorta <2 mm	28 (21)
Dominant ventricular dysfunction greater than moderate	10 (7.5)
Tricuspid regurgitation greater than moderate	12 (9.1)
Restrictive atrial septum^a^	23 (17)

Values are n (%), n, or median (interquartile range).

1 patient did not have gestational age, birth weight, or high-risk cardiac anatomy data.

AA, aortic atresia; AS, aortic stenosis; AV, atrioventricular; DILV, double-inlet left ventricle; DORV, double-outlet right ventricle; HLHS, hypoplastic left heart syndrome; MA, mitral atresia; MS, mitral stenosis; SVCD, single-ventricle cardiac defect; TA, tricuspid atresia.

a Gradient >8mmHg from transthoracic echocardiogram.

Cumulative resource use among the 72 patients who survived to 6 months after the Fontan procedure showed a median procedural LOS of 53.5 days (IQR, 38.0-75.3 days), with 11.5 days interstage LOS (IQR, 6.8-23.3days), 24 cardiology appointments (IQR, 20.0-28.3), 27 echocardiograms (IQR, 22.8-33.3), and 2 cardiac catheterizations (IQR, 1.8-3.0). In patient-years, this translates to a median procedural LOS of 16.2 days (IQR, 11.6-25.2 days), with 3.6 days interstage LOS (IQR, 1.9-7.5 days), 7.4 cardiology appointments (IQR, 6.1-8.5), 8 echocardiograms (IQR, 7.2-10.4), and 0.7 cardiac catheterizations (IQR, 0.4-1.1) per patient-year (Table 2). In contrast, nonsurvivors had a median procedural LOS of 365.3 days (IQR, 342.4-365.3 days) and underwent 56.2 echocardiograms (IQR, 38.3-91.3) per patient-year.

**Table 2:** Indexed Resource Use for the Entirety of the Palliation Pathway

Characteristic	Overall N = 126	Survivors n = 72	Nonsurvivors n = 54
Procedural hospital LOS, d	32 (15-365)	16 (12-25)	365 (365-365)
Interstage admissions	1 (0-2)	1 (1-2)	0 (0-6)
Interstage hospital LOS, d	3 (0-9)	4 (2-8)	0 (0-19)
Cardiology visits	7 (4-9)	7 (6-9)	0 (0-23)
Echocardiograms	11 (8-48)	8 (7-10)	56 (38-91)
Advanced imaging	0 (0-0.3)	0 (0-0.3)	0 (0-0)
Cardiac catheterizations	0.7 (0.3-1.4)	0.7 (0.4-1.1)	0 (0-4)

Median (interquartile range) indexed to patient-years.

LOS, length of stay.

Resource use varied across palliative stages and interstages (Supplemental Tables 1 and 2). HS1 necessitated the lengthiest median hospital LOS (21 days). Resource use peaked during IS1, characterized by frequent echocardiographic monitoring and cardiology evaluations, with nonsurvivors incurring more frequent and lengthier hospitalizations. Comprehensive stage 2 hospitalization had the highest use of advanced imaging and cardiac catheterization (n = 36 [40%] and n = 83 [93%] patients, respectively). During interstage 2, nonsurvivors had more frequent cardiology clinic visits and echocardiograms and more frequent cardiac catheterizations across all stages. Yearly analysis revealed the densest resource use in the first year of life, followed by a substantial and sustained decline thereafter (Supplemental Figure).

**Figure 2:**
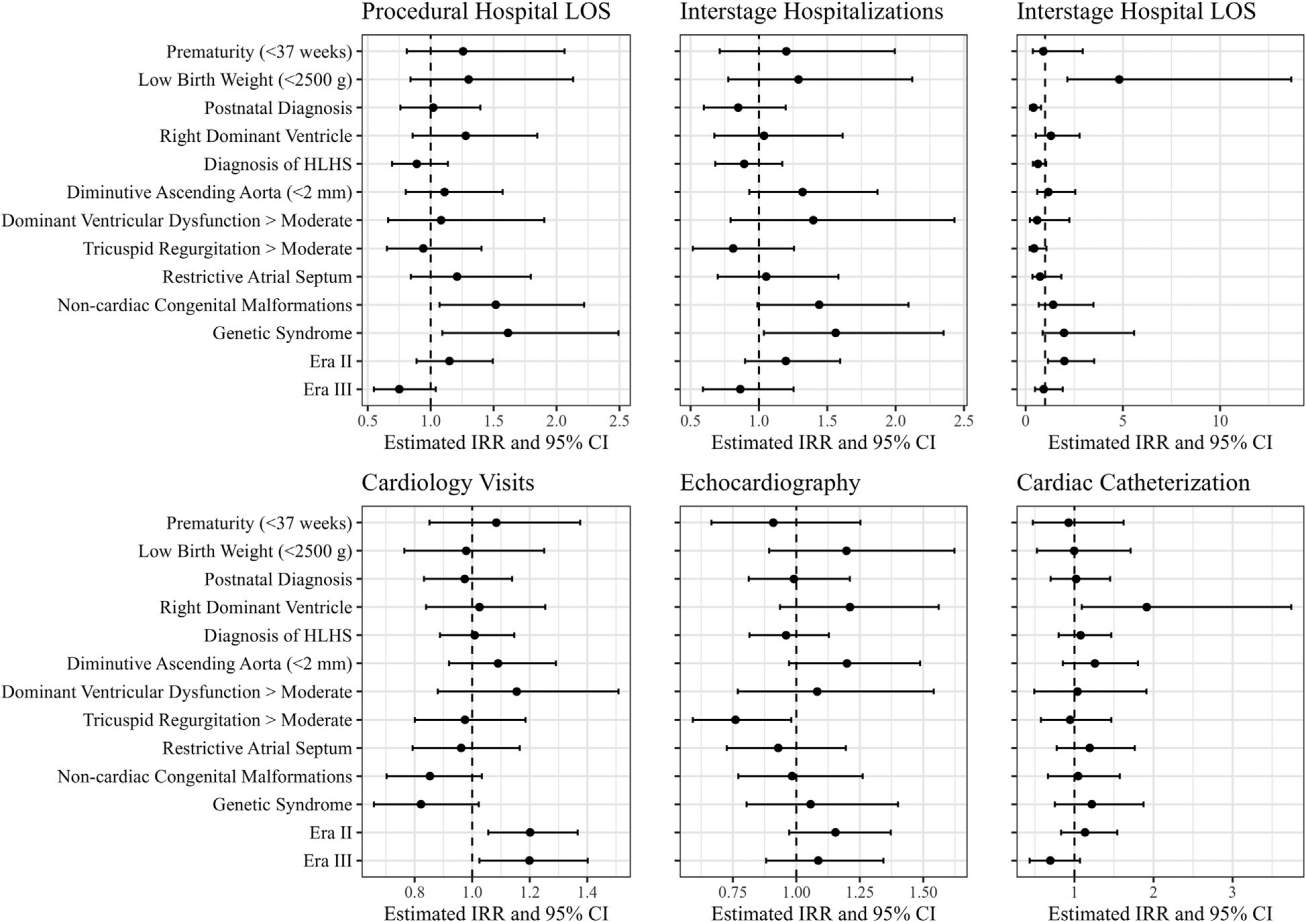
Univariate analysis of select risk factors for increased resource use. Forest plots of select risk factors for increased resource use represent the results of univariable negative binomial regression models. The dots represent the incidence rate ratio (IRR) and the line of 95% CI. The dashed lines indicate the line of no effect (IRR of 1). An IRR less than 1 indicates that the exposure is associated with a lessened rate of event, whereas an IRR greater than 1 indicates that the exposure is associated with an increased rate of event. Era 2: April 2014 to May 2017. Era 3: June 2017 to the present. (HLHS, hypoplastic left heart syndrome; LOS, length of stay.)

Nonsurvivors were more likely to have postnatal diagnoses, restrictive atrial septum, and, although not significant, right ventricular dominance (RVD) and a diminutive aorta (Supplemental Table 3). Compared with their observed times, infants with low birth weight required longer interstage hospitalizations (incidence rate ratio, 4.82; 95% CI, 2.15-13.66). Those patients with RVD underwent a higher rate of cardiac catheterizations (incidence rate ratio, 1.91; 95% CI, 1.09-3.74). Patients with concomitant diagnoses of genetic syndromes experienced higher rates of interstage hospitalizations and LOS (Figure 2).

## Comment

The diagnosis of SVCD compels extensive parental counseling because it is a complex and health care resource–intensive condition.^5^ This report details hybrid palliation–related resource use.

The Society of Thoracic Surgeons reported a median cumulative postoperative LOS of 92.5 days in 2022 for Norwood-to-Fontan palliation.^2^ The Philadelphia Fetus to Fontan study found an overall median cumulative LOS of 57 days.^4^ Our study found a median procedural LOS of 53.5 days, 24 cardiology visits, 27 echocardiograms, and 2 cardiac catheterization procedures through the process of palliation.

The first 6 months after HS1, including IS1, were the most resource intensive (Figure 1). This period is the most vulnerable for patients with SVCDs, necessitating vigilant monitoring.^4^ The median age at death, orthotopic heart transplantation, or Norwood conversion was 116 days (IQR, 37-221 days). Indexed to patient-years, the median LOS of nonsurvivors was 365 days, meaning that these patients spent their entire life in the hospital. Furthermore, nonsurvivors required 3 times the hospitalizations, visits, and catheterizations and 9 times the echocardiograms compared with survivors, findings similar to those in existing literature.^6^ These data underscore the significant health care resource implications tied to mortality, the failure of single-ventricle palliation, and the efforts made to salvage patients with poor outcomes.

Several factors were shown to be associated with increased resource use. Postnatal diagnosis often leads to presentation in extremis, thereby portending higher morbidity and mortality. A total of 21% of our cohort had a postnatal diagnosis. Low birth weight and low operative weight are well-established risk factors associated with increased mortality and morbidity and are common causes of noncardiac admissions in patients with SVCDs.^7^ Our study, encompassing hybrid palliation, also supports this notion.

Furthermore, RVD led to increased cardiac catheterizations, echocardiographs, and procedural LOS, possibly resulting from reduced systolic contractility and eventual heart failure under systemic pressures. This correlation with poor outcomes is well described.^4^ Finally, diminutive ascending aorta and aortic atresia correlated with more visits, echocardiograms, hospitalizations, and catheterizations, albeit not significantly. Because aortic reconstruction is delayed until comprehensive stage 2, coronary and cerebral circulation relies on retrograde perfusion. Thus, obstruction of the retrograde aortic arch is assiduously monitored for and was found to be the most common reason for intervention during catheterization in IS1.

The impact of ventricular morphology and dominance on outcomes cannot be underestimated. RVD and HLHS was present in 93% and 72% of our cohort, respectively. This finding is in contrast to the Philadelphia Norwood cohort, which included only patients with a prenatal diagnosis and had only 41.6% of patients with HLHS. We postulate that this disparity partially accounts for the difference in overall survival (59% vs 68%, respectively). However, our survival rate was on par with more comparable cohorts.^8^

Our cohort included 14% of patients with a diagnosed genetic syndrome, predominantly heterotaxy syndrome. However, genetic syndromes were not predictive of mortality in our analysis. Nonetheless, these patients had higher rates of interstage admissions and longer LOS relative to their contributed time. Both factors had very similar effects on each resource use parameter studied.

As with many programs, we have evolved to a more comprehensive and multidisciplinary approach to such patients. Notable practice changes include establishment of a single-ventricle team, initiation of a digital home monitoring program, implementation of perioperative management protocols aimed at preventing pulmonary artery thrombosis,^5^ introduction of preemptive left pulmonary artery stenting,^9^ and proactive risk mitigation to decreased cardiac arrests for high-risk patients (PROMISE program),^10^ to name a few. These changes have led to a more dependable cardiology follow-up, decreased procedural LOS, and reduced the number of postoperative cardiac arrests.

Limitations of our study include its retrospective design, the relatively low occurrence of certain resources for patients, and the institutional bias toward the hybrid palliation precluding a comparison group. By design, we are unable to provide information on resource use for patients in whom the hybrid pathway failed beyond our designated end points. Additionally, emergency department visits, visits outside of our center, or noncardiac resources used were not captured as part of this study. Our findings are most relevant to other programs that use hybrid palliation.

In conclusion, resource use for the care of patients with SVCDs is significant. The intensity of surveillance is highest in the first year and decreases as patients progress. Protocolization of care and continuous quality improvement lead to improved outcomes and optimization of health care resources. Resource needs and patterns of use in Hybrid palliation are comparable to published Norwood pathway data.

## Figures and Tables

**Supplementary Figure:**