# Supplementary material for: Efficiency at Heart: Navigating the Hybrid Single-Ventricle Pathway
Source: Ann Thorac Surg Short Rep. 2024 Mar 23;2(3):374–9. doi: 10.1016/j.atssr.2024.02.017 (PMC11708584; doi:10.1016/j.atssr.2024.02.017)
Supplement: Supplementary Tables and Figure [file mmc1.docx]

| **Supplemental Table 1.** Resource Utilization of 134 SVCD Patients by Hybrid Palliation Stage | | | | |
| --- | --- | --- | --- | --- |
|  | **Survivors**  Median (IQR) | **Non-Survivors**  Median (IQR) | p-value |  |
|  |  |  |  |  |
| **Hybrid Stage 1** | **n=107** | **n=27** |  |  |
| Hospital LOS | 22 (17, 36) | 32 (13, 95) | 0.419 |  |
| Echocardiograms | 4 (3, 7) | 5 (2, 14) | 0.657 |  |
| Advanced Imaging | 5 (5%) | 3 (11%) | 0.210 |  |
| Cardiac Catheterizations | 8 (7%) | 7 (26%) | **0.006** |  |
| Interventional/total | 6/10 (60%) | 3/9 (33%) |  |  |
| **Comprehensive Stage 2** | **n=88** | **n=7** |  |  |
| Hospital LOS | 12 (8, 23) | 45 (14, 178) | **0.027** |  |
| Echocardiograms | 3 (2, 4) | 3 (3, 9) | 0.143 |  |
| Advanced Imaging | 4 (5%) | 1 (14%) | 0.271 |  |
| Cardiac Catheterizations | 13 (15%) | 3 (43%) | 0.057 |  |
| Interventional/total | 11/19 (58%) | 5/9 (55%) |  |  |
| **Fontan** | **n=72** | **n=2** |  |  |
| Hospital LOS | 12 (9, 19) | 7 (4, 9) | 0.166 |  |
| Echocardiograms | 2 (1, 2) | 3 (1, 4) | 0.943 |  |
| Advanced Imaging | 1 (1%) | 0 (0%) | 0.873 |  |
| Cardiac Catheterizations | 4 (6%) | 1 (50%) | **0.013** |  |
| Interventional/total | 4/5 (80%) | 1/1 (100%) |  |  |
| Data presented for survivors and non-survivors of each stage.  Unadjusted Values presented as median (IQR); For those variables where prevalence was low, data is presented as patient count (%).  LOS, length of stay (days) | | | | |
|  | | | | |

| **Supplemental Table 2.** Indexed Resource Utilization of 107 SVCD Patients in the Hybrid Interstage Periods | | | | |
| --- | --- | --- | --- | --- |
|  | **Survivors**  Median (IQR) | **Non-Survivors**  Median (IQR) | p-value |  |
|  |  |  |  |  |
| **Interstage 1** | **n=95** | **n=12** |  |  |
| Hospitalization count | 3 (0, 6) | 8 (4, 12) | **0.013** |  |
| Hospital LOS | 11 (0, 33) | 60 (21, 128) | **0.007** |  |
| Cardiology Visits | 22 (19, 26) | 16 (12, 23) | 0.099 |  |
| Echocardiograms | 25 (21, 30) | 33 (16, 43) | 0.300 |  |
| Advanced Imaging | 5 (5%) | 2 (17%) | 0.088 |  |
| Cardiac Catheterizations | 48 (51%) | 6 (50%) | 0.651 |  |
| Interventional/total | 39/56 (70%) | 2/9 (22%) |  |  |
| **Interstage 2** | **n=82** | **n=6** |  |  |
| Hospitalization count | 1 (1, 2) | 4 (1, 7) | 0.087 |  |
| Hospital LOS | 2 (1, 5) | 31 (6, 58) | **0.031** |  |
| Cardiology Visits | 4 (3, 5) | 7 (5, 8) | 0.063 |  |
| Echocardiograms | 3 (2, 4) | 8 (5, 13) | **0.018** |  |
| Advanced Imaging | 33 (40%) | 4 (67%) | 0.21 |  |
| Cardiac Catheterizations | 76 (93%) | 5 (83%) | 0.42 |  |
| Interventional/total | 100/134 (75%) | 4/9 (44%) |  |  |
| **6 months post-Fontan** | **n=72** | **n=0** |  |  |
| Hospitalization count | 0 (0, 2) | — | — |  |
| Hospital LOS | 0 (0, 4) | — | — |  |
| Cardiology Visits | 10 (6, 18) | — | — |  |
| Echocardiograms | 2 (0, 4) | — | — |  |
| Advanced Imaging | 10 (14%) | — | — |  |
| Cardiac Catheterizations | 4 (6%) | — | — |  |
| Interventional/total | 5/6 (83%) |  |  |  |
| Data presented for survivors and non-survivors of each stage.  Indexed Values presented as median (IQR) adjusted to per patient-year; For those variables where prevalence was low, data is unadjusted and presented as count (%).  LOS, length of stay (days) | | | | |

| **Supplemental Table 3.** Prevalence of Potential Risk Factors Contributing to Resource Utilization | | | | |
| --- | --- | --- | --- | --- |
|  | Survival (n=79) | Death, OHT, or Norwood (n=55) | P-value |  |
| Prematurity (<37w) | 6 (7.6%) | 7 (16.3%) | 0.33 |  |
| Low Birth Weight (<2500g) | 5 (6.3%) | 8 (18.6%) | 0.12 |  |
| Postnatal diagnosis | 14 (17.7%) | 14 (25.5%) | **0.005** |  |
| Genetic Syndrome | 9 (11.4%) | 11 (20.0%) | 0.26 |  |
| Extracardiac anomaly | 10 (12.7%) | 10 (18.2%) | 0.96 |  |
| Dominant Right Ventricle | 72 (91.1%) | 54 (98%) | 0.095 |  |
| Diminutive Ascending Aorta <2mm | 13 (16.5%) | 16 (29.1%) | 0.082 |  |
| Dominant Ventricular Dysfunction  > Moderate | 4 (5.1%) | 6 (10.9%) | 0.21 |  |
| Tricuspid Regurgitation > Moderate | 9 (11.4%) | 3 (5.5%) | 0.24 |  |
| Restrictive Atrial Septum | 9 (11.4%) | 14 (25.5%) | **0.034** |  |
| OHT, orthotopic heart transplant | | | | |

Supplemental Figure 1: Resource Utilization among 72 Survivors Completing Staged Palliation
